# *orf6* and *orf10* in Prophage phiv142-3 Enhance the Iron-Acquisition Ability and Resistance of Avian Pathogenic *Escherichia coli* Strain DE142 to Serum

**DOI:** 10.3389/fvets.2020.588708

**Published:** 2020-11-25

**Authors:** Dezhi Li, Xinjie Qian, Xinyuan Liu, Yu Sun, Jianluan Ren, Feng Xue, Qing Liu, Fang Tang, Jianjun Dai

**Affiliations:** ^1^School of Medical Instrument and Food Engineering, University of Shanghai for Science and Technology, Shanghai, China; ^2^Ministry of Education Joint International Research Laboratory of Animal Health and Food Safety, Key Laboratory of Animal Bacteriology, Ministry of Agriculture, College of Veterinary Medicine, Nanjing Agricultural University, Nanjing, China; ^3^China Pharmaceutical University, Nanjing, China

**Keywords:** avian pathogenic *Escherichia coli*, prophage, serum resistance, iron acquisition, colonization

## Abstract

Avian pathogenic *Escherichia coli* (APEC), an extraintestinal pathogenic *E. coli* (ExPEC), is the causative agent of avian colibacillosis, a disease that causes huge economic losses in the poultry industry and is characterized by infection through respiratory tract colonization followed by bacteraemia. A previous study in our lab demonstrated that phiv142-3 enhanced the survival ability of APEC strain DE142 in chickens serum. However, the mechanism of this affect has not been completely revealed. Here, we analyzed the transcriptional level of the prophage phiv142-3 region in DE142 when grown in chicken serum. Several upregulated genes attracted our attention, and a series of mutants were constructed. Deletion of *orf6* or *orf10* from phiv142-3 led to lower yields compared with WT after cultivation in serum for 10 h (*P* < 0.05). Furthermore, avian infection assays showed that compared with WT, the bacterial loads in blood and heart tissue of chickens challenged with DE142Δ*orf6* were decreased to 3.9 and 13%, while the bacterial burden in blood and heart from chickens infected with DE142Δ*orf10* was decreased to 7.2 and 8%, respectively (*P* < 0.05). DE142Δ*orf6* showed an obviously attenuated growth rate in the logarithmic phase when cultured in iron-deficient medium, and the transcription level of the *iutA* gene decreased to 43% (*P* < 0.05). The bactericidal assays showed that the survival of the mutant DE142Δ*orf10* was ~60% compared with WT in 50% chicken serum. The K1 capsule-related genes (*kpsF, kpsE, kpsC*, and *kpsM*) were down-regulated nearly 2-fold in DE142Δ*orf10* (*P* < 0.01). Together, these results suggested that *orf6* affects growth by contributing to the uptake ability of iron, while *orf10* increases resistance to serum by upregulating K1 capsule-related genes.

## Introduction

The viruses that infect bacteria, known as bacteriophages (phages), are the most abundant biological entity on Earth, with an estimated global population of 10^31^ (1). They play a crucial role in controlling bacterial populations through phage-mediated killing and the formation of prophages whose DNA inserts into the bacterial genome or is maintained as an episome after infecting bacteria. Most bacterial genomes harbor multiple prophages and contain up to 20% prophage sequences (2, 3). The role of these prophage genes in bacterial genomes remains largely unknown.

In lysogeny, the survival of prophages depends on the survival of the bacterial host in which it resides. Thus, from the perspective of evolution, it is advantageous for prophages to encode genes that promote fitness of the bacterial host. Through comparative genomics, pathogenic strains have been shown to harbor a larger proportion of prophage sequences than non-pathogenic strains (4). The greatest advantage associated with prophages is their ability to increase the resistance of the host to superinfection (5). Moreover, prophages can also alter many traits that increase host survival in different environments (6–8). For instance, it has been reported that after ΦMin27 integrates into the *E. coli* genome, the swimming motility of the lysogen is increased, allowing bacteria to move to high nutrient locations and avoid adverse environments (9). The lysogenic strain *E. coli* MG1655 (ΦMin27) grows faster than MG1655 (without the prophage ΦMin27) (10). A lysogenic *Streptococcus suis* strain (with a prophage integrated into its genome) exhibited a faster growth rate and greater virulence (11). Similarly, Wang knocked out all nine prophages in *E. coli* K-12 BW25113 and found that the resulting strain showed decreased resistance to acid, osmotic and oxidative stresses (12). The Bor and Iss proteins are encoded by a prophage, which conferred resistance to lysogenic *E. coli* (13, 14). Recent studies have also pointed out that prophages increase the competitive fitness of lysogenic hosts and play important roles in bacterial diseases (15–18).

APEC is the causative agent of avian colibacillosis and causes huge economic losses in the poultry industry. The typical route of infection is respiratory tract colonization followed by bacteraemia (19). Resistance to serum appears to be an important factor for APEC infection (20, 21). Studies show that a number of virulence factors, such as outer membrane proteins (OMPs) and capsules, are associated with the complement resistance of *E. coli* (20, 22, 23). In addition, assimilated iron is tightly bound to various proteins in animal blood, so serum is an iron-deficient condition for most bacteria (24, 25). However, iron, which is involved in many bacterial biological processes, is a crucial nutrient for most bacteria (26, 27). This low iron availability is another way that animals are protected against bacterial pathogens. APEC encode four types of siderophores involved in iron uptake systems to obtain iron, including enterobactin, salmochelins, yersiniabactin, and aerobactin. Corresponding receptors on the surface of cell membranes can bind to these sideophores and transfer iron-siderophore complexes into the cell (28).

A previous study in our lab found that prophage phiv142-3 enhanced the survival ability of APEC strain DE142 in chicken serum (29), but the mechanism of this effect is still unclear. The 54 genes in phiv142-3 from attL to attR were named *orf1*-*orf54* according to the order of the locus. Therefore, the goal of this study was 2-fold. First, we aimed to determine which genes in phiv142-3 contribute to bacterial growth in serum and, if any genes were identified, to further characterize these genes. The work presented here described the use of qRT-PCR methods to screen the transcription level of genes in phiv142-3, and two knockout mutants were constructed to identify the role of the genes *in vivo* and *in vitro*.

## Materials and Methods

### Bacterial Strains, Plasmids, Primers, and Culture Conditions

The bacterial strains and plasmids used in this study are summarized in Table 1, and oligonucleotide primers are listed in Table 2. Unless stated otherwise, all *E. coli* cultures were grown at 37°C in Luria-Bertani (LB) liquid medium with aeration. Appropriate antibiotics were added to the LB medium when necessary at the following concentrations: ampicillin (Amp, 100 μg mL^−1^), kanamycin (Kan, 50 μg mL^−1^), and chloramphenicol (Cm, 30 μg mL^−1^).

**Table 1:** Bacterial strains and plasmids used in this study.

**Strains and plasmids**	**Description**	**References**
DE142	APEC strain, O2 serotype	This study
DE142Δ*orf6*	*orf6* deletion mutant	This study
DE142Δ*orf6*/orf6^*^	Complementation strain, containing pSTV28-*orf6* plasmid	This study
DE142Δ*orf6*Δ*iutA*	*orf6* and *iutA* deletion mutant	This study
DE142Δorf6/iutA^*^	Complementation strain, containing pSTV28-*iutA* plasmid	This study
DE142Δ*orf10*	*orf10* deletion mutant	This study
DE142Δ*orf10*/orf10^*^	Complementation strain, containing pSTV28-*orf10* plasmid	This study
DE142Δ*orf24*	*orf24* deletion mutant	This study
DE142Δ*orf40*	*orf40* deletion mutant	This study
DE142Δ*orf41*	*orf41* deletion mutant	This study
DE142A	DE142, containing pUC19 plasmid, Amp	This study
DE142Δ*orf6*C	DE142Δ*orf6*, containing pSTV28 plasmid, Cm	This study
pKD46	Express λ red recombinase, Amp	(30)
pKD4	Template plasmid, Kan	(30)
pCP20 pSTV28	Yeast Flp recombinase gene, FLP, Cm, Amp Expression using lac promotor, Cm	Takara Takara
pUC19	Amp	Takara

**Table 2:** Primers used in this study.

**Primer**	**Sequence (5′ to 3′)**	**Target gene**
K1	CAGTCATAGCCGAATAGCCT	pKD4
K2	CGGTGCCCTGAATGAACTGC	pKD4
Kt	CGGCCACAGTCGATGAATCC	pKD4
pKD46-F	GATACCGTCCGTTCTTTCCTT	pKD46
pKD46-R	TGATGATACCGCCTGCCTTACT	
pCP20-F	ATTGGGTACTGTGGGTTTAGTGGTT	pCP20
pCP20-R	TTGGCTTATCCCAGGAATCTGTC	
*orf6*Mu-F	TTTCACAAATAAGCCGCCAACAA GAAGACGGCATTAACATTAAAAT AATTATATATGATGGTGTAGGCTG GAGCTGCTTC	pKD4
*orf6*Mu-R	AGGTAATAGAATAACCAGATA TGCGGCGCAACGGGTGCTGCG ACTATCTGGAGATTTAACCATA TGAATATCCTCCTTAG	
*orf10*Mu-F	GACCTTTCCTTACTGATGATGA AATAAAAATCATACAAAGTTT TTTAGAGGATGTTGCCAGTGTAG GCTGGAGCTGCTTC	pKD4
*orf10*Mu-R	CATTATCATTTTTTGCATAAT GAATGCATTCATTAGAATGCC TTCCGCCAGGGTTGAAATCATA TGAATATCCTCCTTAG	
*orf24*Mu-F	TACCCCAGTCAGCCAGCGTTAG CGCAGTTAAGCCTTTAACAGCCAT TGTCATTTCCTCTCGTGTAGG CTGGAGCTGCTTC	pKD4
*orf24*Mu-R	AGCGCATTGCCCTGAACAGTATT TGAAGATGGCCAAAGAGGCC AGTGAACAGGAGTGATCCATA TGAATATCCTCCTTAG	
*orf40*Mu-F	AACCTGACATGCGAGAAAGGG CTTCTCCGTCACGGTTTTGTATCG CGGTAAAAACGTTGTGTGTAGGC TGGAGCTGCTTC	pKD4
*orf40*Mu-R	ATTAAGCAGATCATGATTAATTC CTCCCCACAACATTCAAGCTGATAG CGGAGATTAATCCATATGAA TATCCTCCTTAG	
*orf41*Mu-F	ACCTGGATCTGTCTCCCAGGC CCAATCTGTTGCATGTCTGCTC ATGATTAATCTCCGCTAGT GTAGGCTGGAGCTGCTTC	pKD4
*orf41*Mu-R	GATCTTTTTTTGTGTCAGCAC AAAATAACCGTAATCCCAA TACTAATAACAGGGCTTACCC ATATGAATATCCTCCTTAG	
*iutA*Mu-F	CTTCTTTAACTCGCTACACA GCATCTTTGGGCTGATTTTT TCCGCCCGTATGGAGGAATAG TGTAGGCTGGAGCTGCTTC	pKD4
*iutA*Mu-R	CAACTTGGCTGTCAGCGGC CATGTTGATATCAGCGTACCTTT GTTGTAAAGGAATACCGGCATAT GAATATCCTCCTTAG	
RT-*dnaE*-F	ATGTCGGAGGCGTAAGGCT	*dnaE*
RT-*dnaE*-R	TCCAGGGCGTCAGTAAACAA	
RT-*ompA*-F	GGTTAGGTCGTATGCCATA	*ompA*
RT-*ompA*-R	GTCCAGATCGTCAGTGAT	
RT-*bor*-F	TTATTACAGGATGTGCTCAAC	*bor*
RT-*bor*-R	CCCGAAACGAAGAAATGAT	
RT-*kpsF*-F	GGAATGGTGATGGTAGAAGA	*kpsF*
RT-*kpsF*-R	CGTAGCGAATGTCAGAGA	
RT-*kpsE*-F	TCTTATCAGGACAACAACAAC	*kpsE*
RT-*kpsE*-R	CCATCAGCGTATTCACTAAC	
RT-*kpsC*-F	CGTTATGGCGAATGGAAG	*kpsC*
RT-*kpsC*-R	CGTGGCGTCATAGTAGAT	
RT-*kpsM*-F	TAGCAGTATCAGCAATCGT	*kpsM*
RT-*kpsM*-R	AATCAGTGTCTCAAGCAATG	
RT-*fur*-F	ACGTCAGTGCGGAAGATTTAT	*fur*
RT-*fur*-R	GATACCAGCGTCGTCAAACT	
RT-*entA*-F	GAGCGAAAGTTACAGGTTT	*entA*
RT-*entA*-R	GGCAACATCCATCACTTC	
RT-*fepA*-F	GTTGAAGGTCTGGAAGGA	*fepA*
RT-*fepA*-R	AGCATATAAGTGATGTTATTGGT	
RT-*iroA*-F	TTACGGCTGAAGCGGATTAC	*iroA*
RT-*iroA*-R	CCTGGCAGTCACGGTAAATAA	
RT-*iroN*-F	GATATTCAGGTCAACGAT	*iroN*
RT-*iroN*-R	CCAGTTATCTAGCACATAT	
RT-*iucD*-F	TTCTATCGCTTCCTTACA	*iucD*
RT-*iucD*-R	ATACAGGTTATTCATATCTTCA	
RT-*iutA*-F	AGCGTGGTGGCGAATAAA	*iutA*
RT-*iutA*-R	TCCGGTACTCCAGTCAGTATC	
RT-*irp1*-F	GGCGAACCCTGCTATGTATT	*irp1*
RT-*irp1*-R	GTCCATGCAGTACCAGCTAAA	
RT-*irp2*-F	ATGGATGCCTCCAGCTTTAC	*irp2*
RT-*irp2*-R	AAATCATAGCGGGTGTCGATAG	
RT-*fyuA*-F	ATGCCTATGTGGGATGGAATG	*fyuA*
RT-*fyuA*-R	CCAGTCATCGGTGGTGTATTT	

### Sample Preparation and RNA Extraction

The APEC stain DE142 was grown to an OD_600_ of 0.6 in LB broth with or without 100 μM 2,2-dipyridyl (DPD) at 37°C. Then, the total RNA of DE142 was extracted from 3 ml of bacterial culture using the TRIzol reagent (Invitrogen) isolation protocol, and the obtained RNA was purified using an RNeasy Mini Kit (Qiagen). The RNA concentration was determined using a NanoDrop2000 (Thermo Scientific, USA).

### Quantitative Real-Time PCR Assay

The cDNA was amplified by HiScript II Q RT SuperMix for qPCR+gDNA wiper (Vazyme Biotech). According to the instructions of the One Step qRT-PCR SYBR Green Kit (Vazyme Biotech), the mRNA transcription levels were measured. The relative expression levels of the genes were calculated using the 2^−ΔΔCt^ method. Assays were performed three times. The endogenous reference gene *DnaE* was used to analyse the bacterial genes quantitatively.

### Construction of the Knockout Mutant and Complement Strains

The lambda red recombinase system was used to construct the knockout mutants (30) with some modifications. Briefly, the homologous recombination constructs were generated with PCR-purified products with the pKD4 kanamycin resistance gene and 60-nucleotide (nt) homology extensions. The plasmid pCP20 was transferred into the mutant to remove the kanamycin resistance gene. Finally, pCP20 was eliminated by serial subculture in LB at 42°C. The mutants were verified by PCR and sequencing. To construct the complementation strains, the coding sequences of genes and their putative promoter regions were amplified using the DE142 strain as a template, followed by independent cloning into pSTV28-MCS.

### Growth of Strains in Serum, LB, Iron-Deficient, and Iron-Amended Media

Chicken serum was obtained from specific-pathogen-free (SPF) chickens. To determine the growth rate in serum, single colonies of the WT and mutant strains were selected and cultured overnight at 37°C. The bacteria culture were centrifuged and washed twice with PBS, so as to remove the LB media. And then, the OD_600_ values were adjusted to 1.0 with chicken serum; the chicken serum was subcultured (1:20) and incubated at 37°C with shaking at 180 rpm. Assays were performed three times. The optical density at 600 nm of the bacterial culture was measured every 1 h over a period of 16 h.

For growth in LB and iron-deficient media, each bacterial suspension was subcultured in LB with or without 100 μM 2,2-dipyridyl (DPD) according to the proportion of 1:100. Then, the cultures were shaken and incubated at 37°C. Bacterial growth was determined by measuring the OD_600_ values every 1 h over a period of 8 h. Assays were performed three times.

For growth in iron-amended media, 100 μM 2,2-dipyridyl (DPD) was added into LB media and mixed well to make it fully bonded to metal ions. Then 70 μM FeCl_3_ was supplemented to this iron-deficient media. The growth curve was measured by the same method.

### Competition Assays in LB and Iron-Deficient Media

The competition experiments were carried out as previously described (31) with some modifications. For competition assays in LB, *E. coli* strains were grown to log phase, collected and suspended in LB broth. For competition assays in iron-deficient medium, the bacteria were suspended with LB liquid medium supplemented with 100 μM 2,2-dipyridyl. Then, the wild-type strain DE142A was mixed with its derivative strains DE142Δ*orf6*C or DE142Δ*orf* 6/*orf* 6^*^ (1 × 10^8^ CFU for each strain). The mixture was incubated at 37°C for 3 h, and the number of each strain was estimated by counting on LB plates with ampicillin (WT) or chloramphenicol (derivative strains). And plating efficiencies of strains on plates with different antibiotic was measure. The results are shown as the competition index (CI). As the strains were mixed at a 1:1 ratio, the CI represented the value of the number of mutant strains divided by WT. The assay was performed in triplicate with three independent experiments.

### Survival in Chicken Serum

The serum bactericidal assay was performed in a 96-well plate as described previously (32). Briefly, SPF chicken serum was diluted to 50% with PBS. *E. coli* strains grown to log phase were collected and washed twice with ice-cold PBS. A dose of 10 μL each culture suspension (OD_600_ = 1.0) was inoculated into a 96-well plate containing 190 μL of 50 and 100% serum. After incubation for 0.5 h at 37°C, bacterial numbers were calculated using LB plates. The assay was performed in triplicate with three independent experiments.

### Experimental Infection of Chickens via the Air Sacs

The wild-type strain DE142, mutant strains, and complementation strains were cultured in LB until the OD_600_ reached 0.6. After pelleting and washing twice with ice-cold PBS, the bacteria were harvested and resuspended in an appropriate volume of PBS to make the concentration of resuspension reach 2 × 10^8^ CFU.

Forty chickens (7 days old) were divided into five groups and separately challenged with the strains prepared above (2 × 10^7^ CFU for each strain) through the respiratory tract. At 24 h post-infection, birds were sacrificed by CO_2_ asphyxiation. The organs of the birds were harvested and homogenized, and the number of bacteria in the lung, heart and blood was measured by plating dilutions of the bacterial suspensions onto LB plates for counting.

### Ethics Statement

All animal experiments conformed to the guidelines of the Association for Assessment and Accreditation of Laboratory Animal Care International. The animal study protocol was approved by the Ethical Committee for Animal Experiments of Nanjing Agricultural University (SYXK(SU)2011-0036), Nanjing, China.

### Statistical Analysis

All statistical analyses were performed by the GraphPad Prism Software package (GraphPad Software, La Jolla, CA, USA). The Mann-Whitney *U-*test was used to analyse the data from *in vivo* colonization, and the difference in the qRT-PCR data was determined using two-way ANOVA. The rest of the data were analyzed by Student's *t-*test. Figures show mean values. Differences were considered significant at *P* < 0.05 and indicated by an asterisk (^*^).

### Accession Numbers

The protein sequences of ORF6 and ORF10 were submitted to the GenBank database, and the accession numbers were QGF19612.1 and QGF19616.1, respectively.

## Results

### Analysis of Gene Expression Profiles of Prophage phiv142-3 in Chicken Serum and LB *in vitro*

As prophage phiv142-3 increased bacterial survival ability in serum, we analyzed the transcriptional level of the prophage phiv142-3 region in DE142 when grown in chicken serum and LB medium. The results showed that most prophage genes were upregulated in chicken serum, among which *orf6* (putative tail fiber protein), *orf10* (conserved hypothetical protein), *orf24* (hypothetical protein), *orf40* (putative primosomal protein), and *orf41* (putative protein) were the 5 most upregulated genes (Supplementary Table 1).

### *orf6* and *orf10* Increased Cell Growth in Chicken Serum

**Figure 1:**
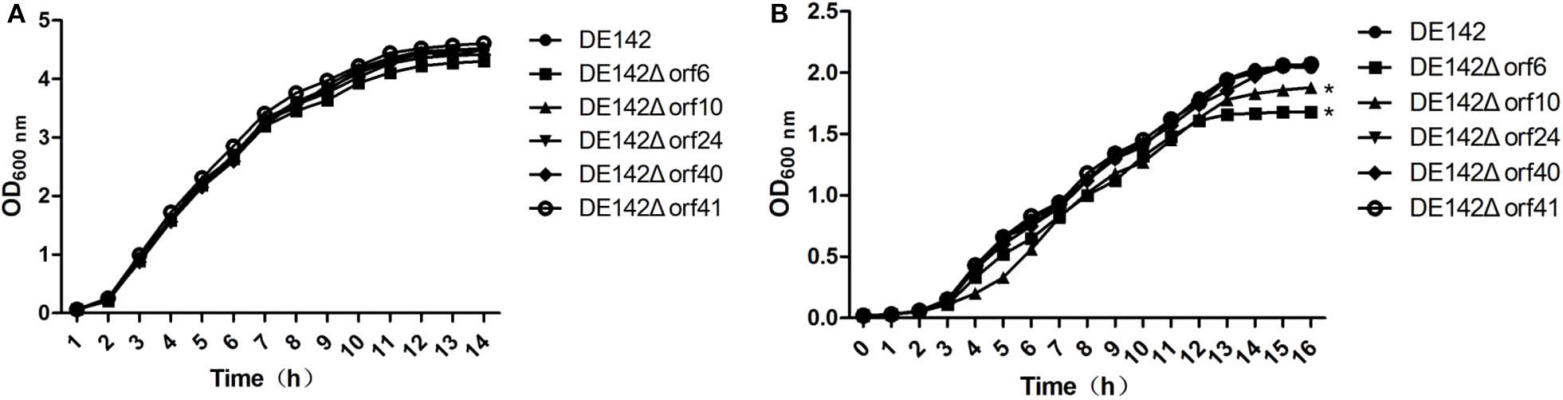
Growth curve of different strains. **(A)** Growth curves of the wild-type strain DE142 and its derivative mutant strains in LB broth. **(B)** Growth curves of the wild-type strain DE142 and its derivative mutant strains in SPF chicken serum. Values were the average of three independent experiments (*P < 0.05 by unpaired *t*-test).

To probe the impact of individual genes in phiv142-3 on bacterial growth in serum, the 5 most upregulated genes mentioned above were knocked out, and the growth of the mutant and WT strains was observed. The deletion mutants showed similar growth curves in LB (Figure 1A). The *orf6* and *orf10* knockout mutants showed a slow growth rate and lower yields on these substrates compared with WT after cultivation in serum for 10 h (*P* < 0.05), while the growth of DE142Δ*orf24*, DE142Δ*orf4*0, DE142Δ*orf41* showed no obvious difference in serum (Figure 1B). Therefore, we surmised that *orf6* and *orf10* conferred the ability of the bacterial strain to grow more rapidly and to obtain higher yields in serum.

### *orf6* Affected the Growth of APEC DE142 in Iron-Deficient Conditions

**Figure 2:**
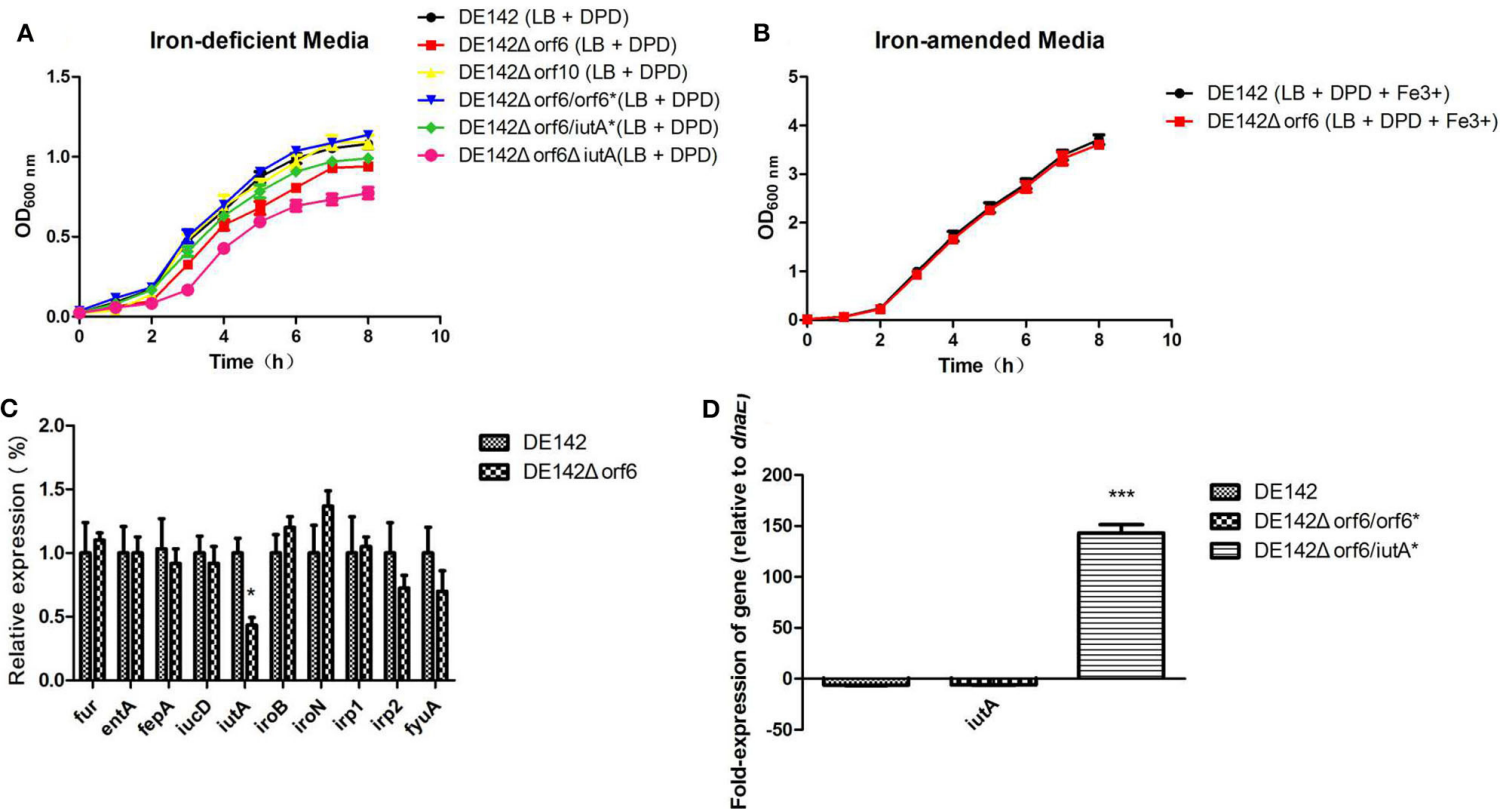
Strain growth in iron-deficient conditions. **(A)** The growth curve of the wild-type strain DE142 and its derivative strains in iron-deficient media (100 μM 2,2-dipyridyl added into LB broth). **(B)** The growth curve of the wild-type strain DE142 and its mutant strains in iron-amended media (70 μM FeCl_3_ was supplemented to iron-deficient media). **(C)** Expression of Fe-acquisition genes. Expression of Fe acquisition genes in the wild-type strain DE142 and the mutant strain DE142Δ*orf6* was measured by RT-PCR. **(D)** Expression of *iutA*. The RNA was extracted from the bacteria grown in iron-deficient media. The acquired cycle threshold (CT) was normalized to the CT of the housekeeping gene *dnaE*. Data were presented as the mean ± SD of three independent experiments, with each experiment being composed of four individual measurements. Unpaired *T*-tests were performed for significance. *P < 0.05, ***P < 0.001.

Next, we tried to investigate the reason for the decreased yields of DE142Δ*orf* 6 and DE142Δ*orf10* in chicken serum. As in animal blood, assimilated iron is tightly bound to various proteins, so serum is an iron-deficient condition for bacteria (33). We speculated that *orf6* and *orf10* might contribute to iron acquisition. To verify our speculation, the growth curves of the mutant and WT strains were analyzed under Fe-deficient conditions. The results showed that the growth rate of the *orf6* deletion mutant was slightly decreased compared with that of WT within 6 h. However, after 6 h, DE142Δ*orf6* showed significantly reduced growth in iron-deficient conditions (*P* < 0.05) compared with WT. And There was no significant difference between the growth rates of WT and DE142Δ*orf10* or DE142Δ*orf6*/orf6^*^ in iron-deficient conditions (Figure 2A). Meanwhile, after FeCl_3_ was added into the iron-deficient media, this difference was disappeared (Figure 2B). Hence, the results indicated that *orf6* contributed to the iron acquisition of DE142 in serum.

To further investigate how *orf6* influences iron acquisition in chicken serum, the transcription levels of well-known genes related to the acquisition of iron, including enterobactin (*entA* and its receptor *fepA*), salmochelin (*iroB* and its receptor *iroN*), yersiniabactin (*irp1, irp2*, and its receptor *fyuA*) aerobactin (*iucD* and its receptor *iutA*) and ferric uptake regulation gene *fur*, were investigated by qRT-PCR. The RNA was isolated from cells grown in iron-deficient medium. Compared with WT, the transcription levels of *iutA* decreased to 43% in DE142Δ*orf6*, while the other genes showed no significant differences between DE142Δ*orf6* and WT (Figure 2C). DE142Δ*orf6*Δ*iutA* showed the slowest growth rate (Figure 2A). However, when obtaining plasmid encoded *iutA* and successful overexpression of *iutA* (Figure 2D), DE142Δorf6/iutA^*^ grew faster than DE142Δ*orf6* but still slower than WT in iron-deficient media (Figure 2A). Meanwhile, the transcription level of *iutA* of DE142Δorf6/orf6^*^ restore to that of WT (Figure 2A). In total, these data indicated that *orf6* not only by upregulating the expression of *iutA*, but also affecting other aspects of the iron-acquisition ability of APEC strain DE142.

### *orf6* Increased Competitiveness in Iron-Limited Conditions

**Figure 3:**
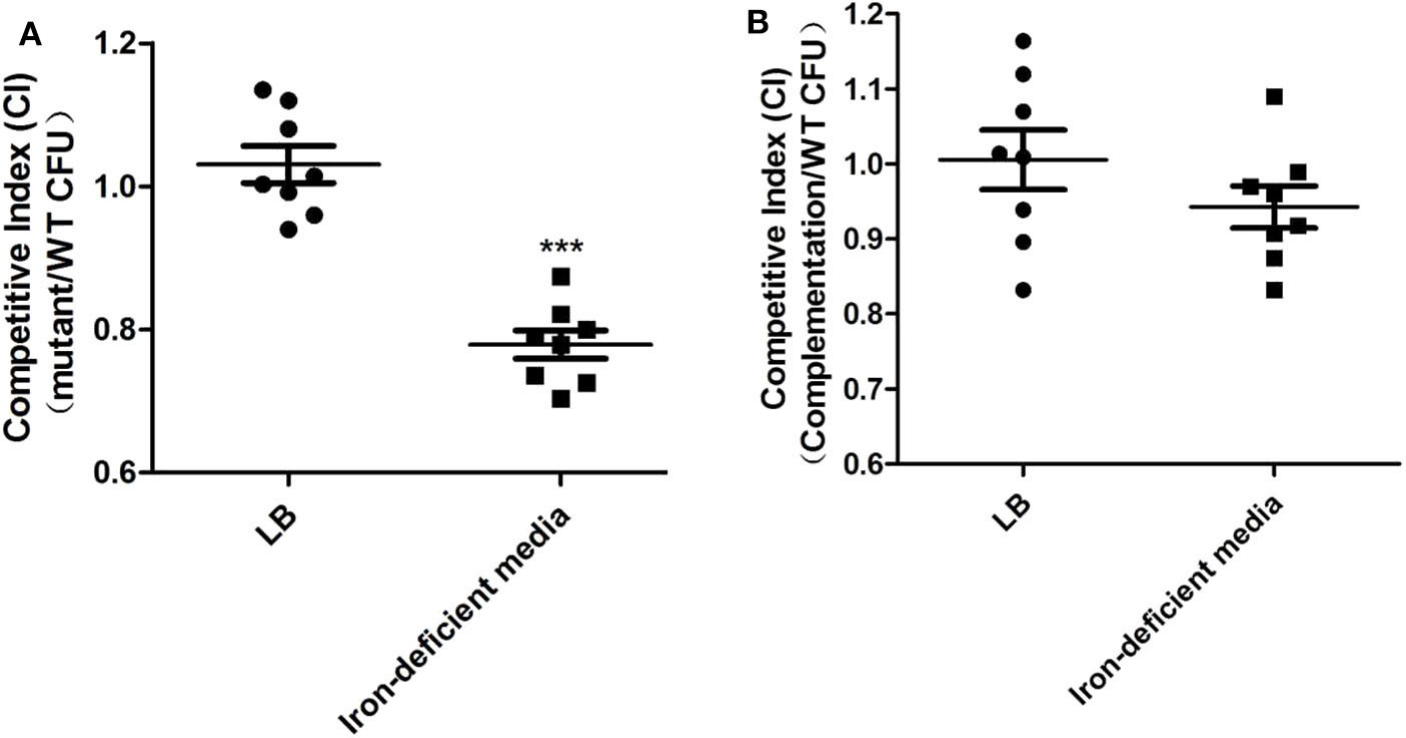
Competition assays. **(A)** The competition between WT and DE142Δ*orf6*. **(B)** The competition between WT and DE142Δ*orf6*/orf6*. Each derivative strain was mixed with the wild-type strain DE142A at a concentration ratio of 1:1. After incubation at 37°C for 3 h, the number of each strain was estimated by counting on LB plates with ampicillin (WT) or chloramphenicol (derivative strains). The competition index (CI) represented the final relative numbers of the derivative strain compared to the wild-type strain DE142A. The Mann-Whitney *U-*test was performed for significance, ***P < 0.001.

The capacity of DE142Δ*orf6* to compete for growth in LB or iron-deficient media *in vitro* was compared with that of WT. In bacterial competition assays, we observed that the strains showed similar cell numbers in LB after incubation for 3 h at 37°C. However, the number of DE142Δ*orf6* cells was ~80% that of WT cells in iron-deficient conditions, with a CI value of 0.78 (Figure 3A, *P* < 0.05). The competitiveness of complementation strain DE142Δorf6/orf6^*^ was restored to those of the WT (Figure 3B).

### The Resistance of the *orf10* KO Mutant to Serum Decreased

**Figure 4:**
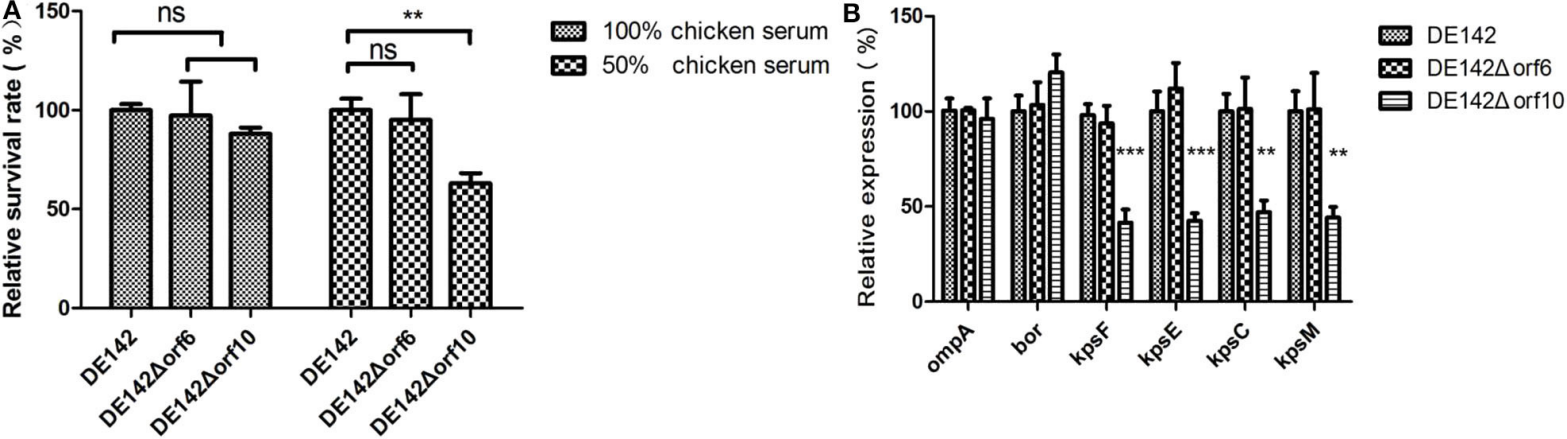
Bacterial resistance to normal chicken serum. **(A)** The survival rate of the strains in chicken serum. Bacteria were incubated with SPF chicken serum at different dilutions at 37°C. After 30 min, the remaining number of surviving bacteria was counted on LB plates. In the 100% chicken serum, the 100% repesents a 18% of inoculum of the WT; While in the 50% chicken serum, the 100% repesents a 32% of inoculum of the WT. DE142Δ*orf10* showed significantly reduced resistance in 50% chicken serum. One-way analysis was performed for significance. **P < 0.01. **(B)** Quantification of genes related to serum resistance. Expression levels of *ompA, bor* and K1 capsule-related genes (*kpsF, kpsE, kpsC*, and *kpsM*) in DE142 (WT) and mutant strains were measured by qRT-PCR. The acquired cycle threshold (CT) was normalized to the CT of the housekeeping gene *dnaE*. Data were presented as the mean ± SD of three independent experiments, with each experiment being comprised of four individual measurements. Unpaired *T*-tests were performed for significance. **P < 0.01, ***P < 0.001.

Next, we investigated the function of *orf10* in serum survival. Assays designed to measure the bactericidal activity of mutant and WT cells in serum were performed. The bactericidal assays showed that the survival of the mutant DE142Δ*orf10* was ~60% (*P* < 0.01) compared with WT in 50% chicken serum. The results also showed that there were no significant differences in 50 and 100% chicken serum between WT and DE142Δ*orf6* (Figure 4A). These results suggested that *orf10* enhances the resistance of DE142 to serum.

To further illustrate how *orf10* enhances the resistance of DE142 to serum, the transcription levels of several well-known genes related to serum resistance in *E. coli (ompA, bor*, and K1 capsule-related genes *kpsF, kpsE, kpsC*, and *kpsM)* (20) were measured by qRT-PCR. RNA was extracted from bacteria growing in LB. The results showed that these genes, except for *bor* and *ompA*, were significantly downregulated in DE142Δ*orf10* (*P* < 0.01). The mRNA levels of these genes showed no significant differences between DE142Δ*orf6* and WT (Figure 4B). This result suggested that the *orf10* gene enhanced bacterial resistance to serum by upregulating the expression of K1 capsule-related genes.

### *orf6* and *orf10* Contributed to Colonization in Chicken Blood and Heart

**Figure 5:**
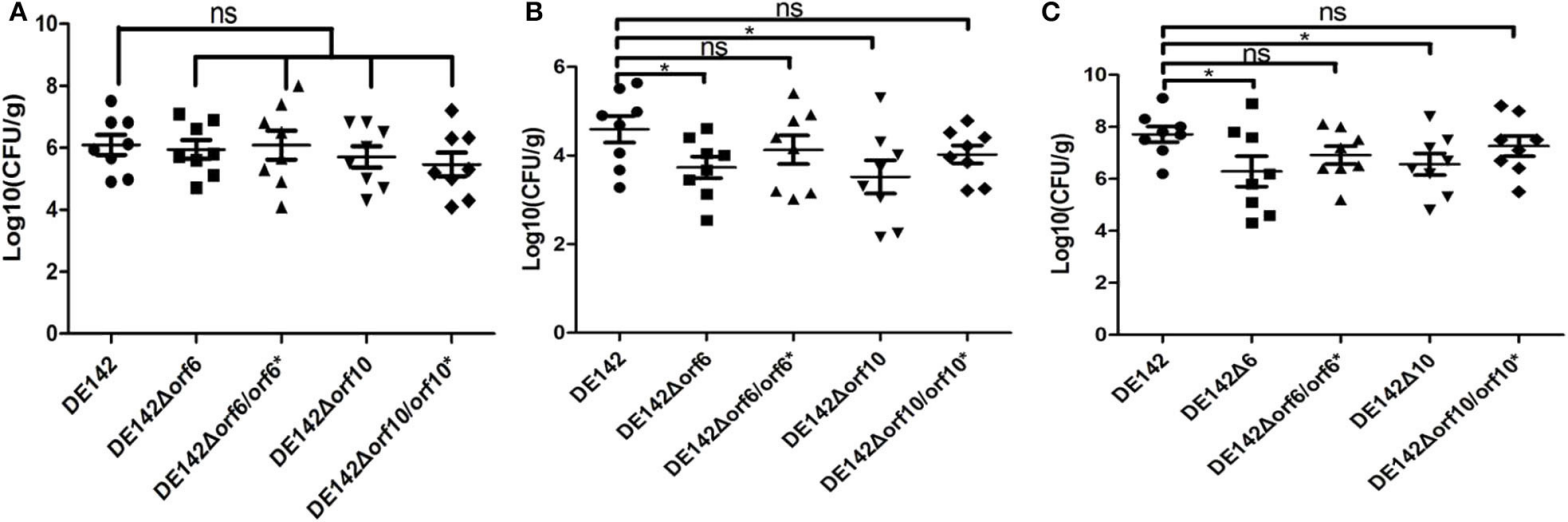
Bacterial colonization during infection *in vivo*. Infection of chickens via the air sacs with 2 × 10^7^ CFU of WT DE142, DE142Δ*orf6*, DE142Δ*orf6*/orf6*, DE142Δ*orf10*, or DE142Δ*orf10*/orf10*. After 24 h post-infection, bacterial re-isolation from the **(A)** lung, **(B)** heart, and **(C)** blood was quantified by the plate counting method. Each data point represented a sample from an individual chicken. The Mann-Whitney *U*-test was performed for significance, *P < 0.05.

To determine whether *orf6* and *orf10* play a role in serum survival *in vivo*, animal experiments were carried out to measure the colonization ability of the WT and its derivative strains. Compared with those challenged with WT, the bacterial loads in blood and heart tissue of chickens challenged with DE142Δ*orf6* were decreased to 3.9 and 13%, while the bacterial burden in blood and heart from chickens infected with DE142Δ*orf10* was decreased to 7.2 and 8%, respectively (Figures 5B,C). There were no significant differences in the lung (Figure 5A). The loads of the complementation strain were restored to those of the WT. The results indicated that *orf6* and *orf10* indeed contributed to the survival of D142 in chicken serum *in vivo*.

## Discussion

APEC strains cause infection, which leads to huge economic losses in the poultry industry worldwide (34). The ability of APEC strains to resist serum may play an important role in the pathogenesis of avian colibacillosis (21). Thus, improving the understanding of the mechanisms of bacterial resistance to serum would be beneficial to the poultry industry. A previous study in our laboratory revealed that knocking out prophage phiv142-3 from DE142 resulted in a decreased survival rate of the mutant strain in chicken serum (29). In this study, we screened the transcription level of phiv142-3 genes in cells grown in serum and LB by qRT-PCR. Then, several phiv142-3 genes were upregulated when cultured in chicken serum and selected for investigation. Our results indicated that *orf6* and *orf10* increased DE142 growth in serum by promoting the acquisition of iron and increasing bacterial resistance to serum, respectively.

In order to identify the genes in phiv142-3 associated with bacterial growth in serum, the transcription level of bacterial genes in LB were compared. Some genes (14/54) in phiv142-3 were significantly upregulated when cells were cultured in chicken serum (Supplementary Table 1). The phiv142-3 genes that were significantly upregulated, *orf6* (2^2.37^-fold), *orf10* (2^4.12^-fold), *orf24* (2^1.25^-fold), *orf40* (2^3.68^-fold), and *orf41* (2^4.56^-fold), were selected, and whether they play roles in bacterial growth in serum was tested. Their different mutants were constructed.

**Figure 6:**
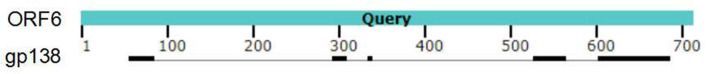
Analyses of the sequences of ORF6 and gp138. Identical regions were highlighted in black.

First, we observed that the *orf6* and *orf10* knockout mutants showed a slower growth rate than the WT in chicken serum, and deletion of orf6 or orf10 didn't influence the transcription of the downstream genes nearby (data not shown). After cultivation in serum for 10 h, the two mutants obtained lower yields (*P* < 0.01) on these substrates compared with WT (Figure 1B). However, there were no significant differences in growth among the WT and its mutant strains when cultured in LB (Figure 1A). In animal blood, assimilated iron is tightly bound to various proteins, so serum is an iron-deficient condition for APEC (28). Thus, we examined the growth curves of strains in iron-deficient conditions. As shown in Figure 2A, the logarithmic growth phase of the *orf6* deletion mutant was significantly decreased compared with that of the WT and the complement strain DE142Δorf6/orf6^*^ was restored to that of WT. Combination with the result in Figure 2B, after FeCl_3_ was added into the iron-deficient media, DE142Δ*orf6* and WT showed a similar growth rate. It indicated that orf6 affecting the iron-acquisition ability of WT. Next, we determined the transcription level of genes related to the acquisition of iron. The mRNA levels of *entA, fepA, iroN, irp1, irp2, fyuA, iucD*, and *fur* in DE142Δ*orf6* showed no significant difference from those in WT. In DE142Δ*orf6*, the transcription levels of *iutA* decreased to 43% (Figure 2C, *P* < 0.05). The iutA protein, which is present on the surface of the cell, is the receptor of the aerobactin siderophore (35). When transfered the plasmid encoding *orf6* into DE142Δ*orf6* resulting in a rise of the transcription level of *ituA* and growth rate in iron-deficient media (Figures 2A,D). Here, the deletion of *orf6* lead to the decreased expression of *iutA* and decrease the iron uptake ability of DE142Δ*orf6*. As the overexpresion of *iutA* in DE142Δorf6/iutA^*^ still grew slower than WT in iron-deficient media, and We speculated that *iutA* was an important but not the only reason that *orf6* affected the iron-acquisition ability of APEC strain DE142. Furthermore, the interaction between bacteria and the host and the competition for iron are crucially important for the outcome of infection (28). The competition assays also showed that DE142Δ*orf6* was at a disadvantage when incubated with DE142 in iron-deficient medium (Figure 3). To our knowledge, no study has demonstrated that prophage and iron acquisition are directly connected, but this connection could be mediated by a quorum-sensing system. By sensing available iron in the environment and producing quorum-sensing signals, bacteria can adjust their iron acquisition ability. For instance, after obtaining a plasmid containing a functional copy of LuxS, an *E. coli* strain achieved high cell density under iron-limited conditions (36). Meanwhile, the prophages phiCDHMI and phi3T have been demonstrated to produce quorum sensing precursors (37, 38). On the other hand, recent reports have revealed that the tail protein gp138 from bacteriophage φ92 contains an iron-binding region (39), and the ORF6 protein in this study has 24% query coverage with gp138 by blastp on NCBI (Figure 6). The expression of two CJIE1 prophage tail proteins has been reported to be upregulated in sheep blood (40), indicating that prophage tail proteins may play a role in bacterial growth in animal blood. Here, ORF6 is a putative tail fiber protein based on genome annotation. Whether ORF6 acts as a “siderophore” or influences iron acquisition by affecting quorum-sensing signals needs to be further studied.

The bactericidal assays performed with SPF chicken serum revealed that the resistance of DE142Δ*orf10* to serum was decreased. The K1 capsule is reported to participate in bacterial resistance to serum (23). Here, K1 capsule-related genes were found to be downregulated nearly 2-fold in DE142Δ*orf10* (Figure 4B). These results were consistent with the previous observation that DE142Δ*orf10* showed a slower growth rate when grown in serum at first, and as time passed, the mutant obtained lower yields on these substrates compared with WT after cultivation in serum for 12 h (Figure 1B, *P* < 0.01). Paniagua-Contreras, G. L found that the Lambda prophage in *E. coli* could increase host bacteria survival ability in human serum (41). Prophages not only encode proteins directly participating in serum bactericidal effects but also alter the traits of their host bacteria (7). Here, *orf10* contributed to serum resistance by influencing the expression of K1 capsule-related genes.

APEC invades the host through the respiratory tract, followed by bacteraemia; thus, serum resistance appears to be an important factor for APEC infection (21). *In vivo* studies revealed that the ability of the mutant strains DE142Δ*orf6* and DE142Δ*orf10* to colonize chicken heart and blood was significantly decreased compared with that of the WT strain (Figures 5B,C). These results corresponded with the *in vitro* observations that DE142Δ*orf6* and DE142Δ*orf10* obtained lower yields when cultured in chicken serum (Figure 1B). However, *orf6* and *orf10* did not affect the bacterial colonization ability in the lung (Figure 5A) or adhesion ability to DF-1 cells (data not shown). The results indicated that *orf6* and *orf10* did not affect bacterial adhesion ability but played a role in bacterial resistance to serum *in vivo*.

In this study, some genes in phiv142-3 were screened and identified to be associated with bacterial growth in serum. Deletion of the prophage genes *orf6* and *orf10* led to a decrease in the number of bacteria when incubated with serum and decreased the ability of the bacterial strain to colonize chicken blood and heart tissue. Our results suggested that *orf6* affects these aspects by improving the uptake ability of iron, while *orf10* increased serum resistance by upregulating K1 capsule-related genes.

## Conclusion

The present work investigated the roles of genes in prophage phiv142-3 associated with bacterial growth in serum *in vitro* and *in vivo*. Through qRT-PCR, *orf6*, and *orf10* in phiv142-3 attracted our attention because they were upregulated in chicken serum compared with LB. The *orf6* and *orf10* knockout mutants showed a slower growth rate than WT in chicken serum. Meanwhile, compared with WT, the bacterial loads in blood and heart tissue of the chickens challenged with DE142Δ*orf6* or DE142Δ*orf10* were also significantly decreased *in vivo*. Furthermore, we explored the deeper reason for these phenomena. On the one hand, the growing ability of DE142Δ*orf6* was attenuated in iron-deficient media. Further study found *iutA* was an important but not the only reason that *orf6* affected the iron-acquisition ability of APEC strain DE142. On the other hand, bactericidal assays performed with SPF chicken serum revealed that the resistance of DE142Δ*orf10* to serum was decreased. The K1 capsule is reported to participate in bacterial resistance to serum. Here, K1 capsule-related genes were found to be downregulated nearly 2-fold in DE142Δ*orf10*. Thus, we speculate that *orf6* affects APEC strain DE142 growth in serum by improving the uptake ability of iron, while *orf10* increases the resistance of DE142 to serum by upregulating K1 capsule-related genes.

## Data Availability Statement

The protein sequences of ORF6 and ORF10 were submitted to the GenBank database, 198 and the accession numbers were QGF19612.1 and QGF19616.1, respectively.

## Ethics Statement

All animal experiments conformed to the guidelines of the Association for Assessment and Accreditation of Laboratory Animal Care International. The animal study protocol was approved by the Ethical Committee for Animal Experiments of Nanjing Agricultural University (SYXK(SU)2011-0036), Nanjing, China.

## Author Contributions

JD concevied and designed the study. DL was responsible for experimental operation and drafted the manuscript. XQ, XL, and YS gave experimental help. JR, FX, and QL gave article modification help. FT provided valuable suggestions of the manuscript. All authors approved the final version of the manuscript.

## Conflict of Interest

The authors declare that the research was conducted in the absence of any commercial or financial relationships that could be construed as a potential conflict of interest.
