# Supplementary material for: orf6 and orf10 in Prophage phiv142-3 Enhance the Iron-Acquisition Ability and Resistance of Avian Pathogenic Escherichia coli Strain DE142 to Serum
Source: Front Vet Sci. 2020 Nov 25;7:588708. doi: 10.3389/fvets.2020.588708 (PMC7724020; doi:10.3389/fvets.2020.588708)
Supplement: Supplementary file 1 [file Table_1.DOC]

**TABLE S1| Significantly up-regulated genes**

| Gene locus | Gene name | Function of product | Log_2_ ratio (in serum/in LB) |
| --- | --- | --- | --- |
| L2sta_01256 | *orf2* | putative endolysin | 1.33 |
| L2sta_01260 | *orf6* | putative tail fiber protein | 2.37 |
| L2sta_01262 | *orf8* | putative anti-repressor protein | 1.31 |
| L2sta_01264 | *orf10* | conserved hypothetical protein | 4.12 |
| L2sta_01278 | *orf24* | hypothetical protein | 1.25 |
| L2sta_01280 | *orf26* | putative tail protein | 1.60 |
| L2sta_01287 | *orf33* | eaa protein | 1.37 |
| L2sta_01291 | *orf37* | putative protein | 1.94 |
| L2sta_01293 | *orf39* | putative replication protein P | 1.91 |
| L2sta_01294 | *orf40* | putative primosomal protein | 3.68 |
| L2sta_01295 | *orf41* | putative protein | 4.56 |
| L2sta_01299 | *orf45* | putative protein | 1.80 |
| L2sta_01301 | *orf47* | exonuclease VIII/RecE-like protein | 1.46 |
| L2sta_01302 | *orf48* | recT family protein | 1.33 |
